# A Case of Unresectable Papillary Thyroid Carcinoma Treated with Lenvatinib as Neoadjuvant Chemotherapy

**DOI:** 10.1155/2020/6438352

**Published:** 2020-05-11

**Authors:** Hiroyuki Iwasaki, Soji Toda, Hiroyuki Ito, Daiji Nemoto, Daisuke Murayama, Yoichiro Okubo, Hiroyuki Hayashi, Tomoyuki Yokose

**Affiliations:** ^1^Department of Breast and Endocrine Surgery, Kanagawa Cancer Center, Yokohama, Japan; ^2^Department of Respiratory Surgery, Kanagawa Cancer Center, Yokohama, Japan; ^3^Department of Pathology, Kanagawa Cancer Center, Yokohama, Japan; ^4^Department of Pathology, Yokohama City Hospital, Yokohama, Japan

## Abstract

A 75-year-old woman visited a nearby clinic with complaints of right clavicle discomfort, and she underwent diagnostic thoracoscopic lung biopsy, being diagnosed with lung metastasis and a right-upper mediastinal mass. The superior mediastinum mass was extrapulmonary and covered by the pleura, and it was not biopsied. Papillary thyroid carcinoma was diagnosed following biopsy of the lung metastasis. Only a small tumor, with a maximum diameter of 70 mm from the right neck to the superior mediastinum, in the thyroid gland invades the internal jugular vein and subclavian vein, forming a tumor embolus in the right brachiocephalic vein and reaching the vicinity of the superior vena cava. For life-saving purposes, we obtained approval from the Cancer Board of Kanagawa Cancer Center and used lenvatinib according to unresectable undifferentiated cancer IRB approval number 28–41. The tumor had shrunk after 4 months, and surgery was performed. The postoperative course has been good, and the patient is being followed up. The patient is alive three months after surgery, and lung metastases have disappeared on CT images. This case is reported as a successful case of neoadjuvant chemotherapy and interval debulking surgery.

## 1. Introduction

Lenvatinib has been recognized to be effective against radioactive iodine (RAI) refractory-differentiated thyroid carcinoma following disease progression [1]. It has also been approved for the treatment of anaplastic thyroid cancer in Japan, and clinical results have been reported [2, 3]. The neoadjuvant approach to BRAF-mutated anaplastic thyroid cancer (ATC) has been reported as clinical trials [4, 5]. Still, there are no reports for the use of neoadjuvant chemotherapy (NAC) + interval debulking surgery for patients with unresectable differentiated thyroid cancers (DTC).

## 2. Case Presentation

**Figure 1:**
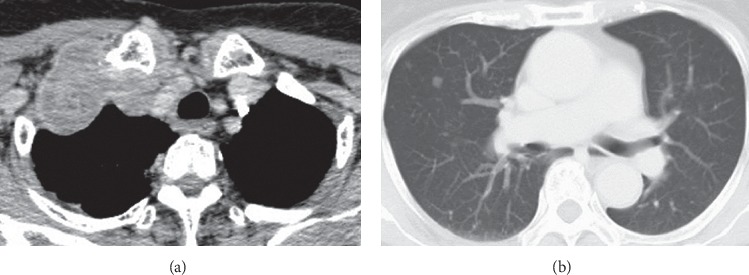
Images taken during the initial visit. Computed tomography revealed a large right-upper mediastinal tumor (a) and micropulmonary metastases (b).

**Figure 2:**
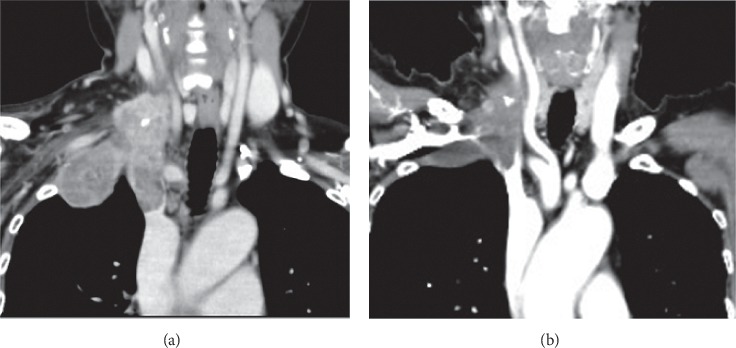
Images taken at baseline and 14 weeks after lenvatinib treatment. The tumor shrank upon treatment, and the major axis was reduced in diameter from 68 to 48 mm. The tumor embolus regressed from the junction of the superior vena cava.

The case report involved a 75-year-old woman with no special medical history. The patient's height and weight were 147 cm and 49 kg, respectively. She visited a nearby clinic and presented with a tumor on the right clavicle and a sense of discomfort. Computed tomography (CT) revealed a large right-upper mediastinal tumor and micropulmonary metastases (Figures 1(a) and 1(b)). The large tumor extended to the neck, subclavicle, and right-upper mediastinum. Partial resection of the lung metastasis was performed under thoracoscopy to diagnose the primary tumor. We did not biopsy the superior mediastinal tumor because it was an extrapulmonary lesion with no continuity with the lung lesion, which was covered by pleura. The pathological diagnosis was lung metastasis of papillary thyroid carcinoma. Although a small mass was also found in the thyroid gland, there was no malignant finding, and treatment was prioritized without further examination. Because the tumor obstructed the right brachiocephalic vein and approached the superior vena cava (SVC) junction, there was a risk of SVC occlusion and pulmonary embolism (Figure 2(a)). We decided to start treatment as soon as possible to avoid sudden death from pulmonary embolism. Lenvatinib treatment was started with approval from the Cancer Board of Kanagawa Cancer Center (IRB approval number 28–41). The tumor shrank upon treatment, and the major axis was reduced from 68 to 48 mm in diameter. The tumor embolus regressed from the junction of the SVC, permitting the right brachiocephalic vein to be clamped and ligated safely (Figure 2(b)).

### 2.1. Treatment Progress

**Figure 3:**
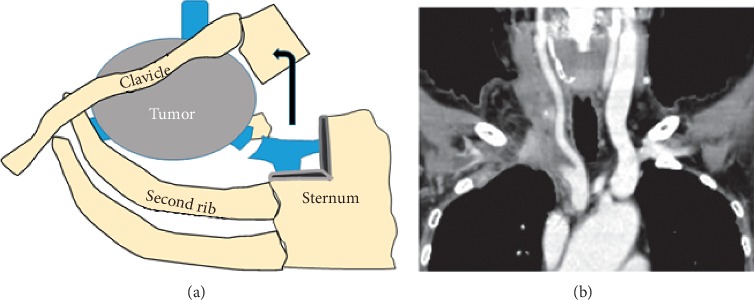
Surgical procedure and after surgery image ((a) and (b)). The sternum was incised as shown in the figure; the sternoclavicular joint and collarbone were flipped outward, and surgery was performed.

The adverse events induced by lenvatinib included grade 2 HT, grade 2 hand-foot syndrome, and grade 2 anorexia, and thus, the dose was reduced to 10 mg after 3 weeks. After 16 weeks of treatment, her weight loss reached 10 kg, and it was clinically assessed that further continuation of lenvatinib was impossible. Then, surgery was performed 1 week after withdrawal of lenvatinib. Total thyroidectomy and resection of the first rib, right internal jugular vein, right subclavian vein, and tumor were performed, and the right brachiocephalic vein was clamped and separated from the SVC junction at the periphery. Infiltration of the subclavian artery and vagal nerve was found on the dorsal side of the tumor, and a tumor approximately 1 cm^2^ in size remained at the site. The risk of SVC syndrome and pulmonary embolism was resolved, and the tumor was largely resected.  Figure 3 shows the schema of the surgical procedure and CT after the operation. The sternum was incised as shown in the figure, the sternoclavicular joint and collarbone were flipped outward, and surgery was performed. We planned to conduct follow-up of the efficacy of RAI therapy and thyroid stimulating hormone (TSH) suppression for small residual tumors and lung metastases.

### 2.2. Pathological Findings

**Figure 4:**
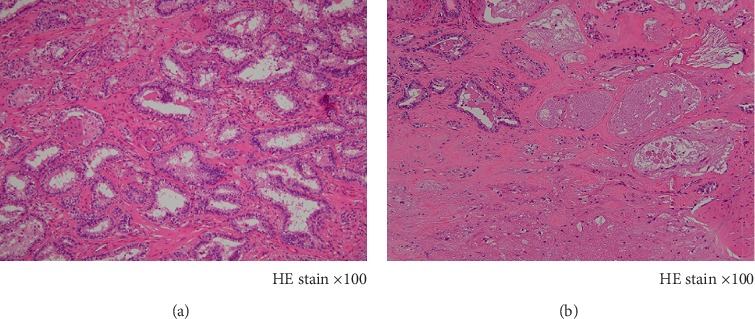
Histologic sections of resected tumor ((a) and (b)). (a) Histopathological image of the central part of the papillary carcinoma tumor. The tumor formed a papillary structure. Individual cancer cells had nuclear grooves, and findings suggestive of nuclear inclusions were also observed. Original magnification 100×. (b) Histopathological image of the periphery of the tumor. Extensive necrosis and fibrosis caused by lenvatinib were observed. Original magnification 100×.

No primary lesion remained in the thyroid gland, and the lenvatinib had a beneficial effect on papillary carcinoma originating from the upper mediastinum.  Figure 4(a) presents a histopathological image of the central region of the papillary carcinoma lesion.  Figure 4(b) presents a histopathological image of the periphery of the tumor. Extensive necrosis and fibrosis caused by lenvatinib were observed. Treatment efficacy was clearly recognized. The patient's preoperative thyroglobulin and TgAb levels were 4690 ng/mL and 18 IU/mL, respectively, decreasing to 451 ng/mL and <10 IU/mL, respectively, after the operation.

## 3. Discussion

### 3.1. Choice of Treatment

In this case, if the tumor had progressed without treatment, obstruction of the SVC would have led to SVC syndrome, and once the embolus flowed to the pulmonary artery, there would have been a risk of sudden death from pulmonary embolism. In fact, micropulmonary metastasis was recognized because a small tumor embolus reached the lung and caused distant metastasis. Under these conditions, surgical resection is extremely risky, and radiotherapy cannot reduce the risk of tumor embolism. Lung metastasis is a differentiated thyroid carcinoma in the histological diagnosis; thus, a good prognosis can still be expected if the lesion can be resected, and RAI therapy can be performed after total thyroidectomy.

### 3.2. Selection of Dose and Timing of Interval Debulking Surgery

The initial dose of lenvatinib was set as 14 mg because of the possibility of tumor embolism due to the rapid tumor disintegration and bleeding caused by the collapse of large blood vessels. We previously reported that the antitumor effect is expected to be the same even at low doses [6]. Adverse events and treatment progress were described. As previously reported [7], surgery should be performed at the time when tumor reduction stops after 3 months of treatment because anaplastic transformation or regrowth may occur.

### 3.3. Withdrawal Period and Problems during and after Surgery

According to the pharmacokinetics of lenvatinib [8], the half-life is 34.5 h, which means that the half-life reached approximately five times in 7 days, meaning that the initial dose had been reduced by 32-fold. Previous experimental data indicated that a 7 day withdrawal had no therapeutic effect on VEGF inhibition, and regrowth was observed [9]. Thus, surgery was performed on day 7. At the time of surgery, adhesion was around the tumor only at the invaded site, and resection was performed as a normal surgical operation. Our greatest concern was delayed wound healing. Despite our concerns, healing was normal. A large tumor was removed, and the right cephalic vein, apical pleura, and first rib were also removed. Therefore, postoperative leachate retention continued for approximately 1 month, which was not sufficient for drainage. Edema of the right-upper limb was observed for approximately 1 week, after which it was resolved. No upper limb movement limitation or paresthesia was observed. The patient is alive three months after surgery, and lung metastases have disappeared on CT images so far. In the future, if NAC + surgery can be safely selected even for patients with difficult resection, it may be possible to improve the treatment results of advanced thyroid cancer.

## Data Availability

The datasets used and/or analyzed during the current study are available from the corresponding author upon reasonable request.
